# Clinical indications for cesarean delivery among women living with female genital mutilation

**DOI:** 10.1002/ijgo.12234

**Published:** 2017-07-20

**Authors:** Maria I. Rodriguez, Lale Say, Jasmine Abdulcadir, Michelle J. Hindin

**Affiliations:** ^1^ Department of Obstetrics and Gynecology Oregon Health and Science University Portland OR USA; ^2^ Department of Reproductive Health, and Research World Health Organization Geneva Switzerland; ^3^ Department of Obstetrics and Gynaecology Geneva University Hospitals Faculty of Medicine University of Geneva Geneva Switzerland; ^4^ Department of Population, Family and Reproductive Health The Johns Hopkins Bloomberg School of Public Health Baltimore MD USA

**Keywords:** Cesarean delivery, Female genital mutilation

## Abstract

**Objective:**

To compare primary indications for cesarean delivery among patients with different female genital mutilation (FGM) status.

**Methods:**

The present secondary analysis included data from women who underwent trial of labor resulting in cesarean delivery at 28 obstetric centers in six African countries between November 1, 2001, and March 31, 2003. Associations between cesarean delivery indications and FGM status were assessed using descriptive statistics and multivariable multinomial logistic regression.

**Results:**

Data from 1659 women (480 patients with no type of FGM and 1179 patients with FGM [any type]) were included; cesarean delivery indications were collapsed into five categories (fetal indications, maternal factors, stage 1 arrest, stage 2 arrest, and other). The incidence of a clear medical indication for cesarean delivery did not differ between the groups (*P*=0.320). Among patients without a clear indication for cesarean delivery, women with FGM were more likely to have undergone cesarean delivery for maternal factors (adjusted relative risk ratio [aRRR] 3.92, 95% confidence interval [CI] 1.3–11.71), stage 1 arrest (aRRR 7.74, 95% CI 1.33–45.07), stage 2 arrest (aRRR 6.63, 95% CI 3.74–11.73), or other factors (aRRR 2.41, 95% CI 1.04–5.60) rather than fetal factors compared with women who had no type of FGM.

**Conclusion:**

Among women with unclear medical indications, FGM was associated with cesarean delivery being performed for maternal factors or arrest disorders.

## Abstract 2

Women living with FGM were more likely to have maternal factors of arrest disorders contribute to undergoing a cesarean delivery for an unclear medical indication.

## 1 INTRODUCTION

Female genital mutilation (FGM) includes procedures involving partial or total removal of the external female genitalia for non‐therapeutic reasons.^1^ WHO has defined four types of FGM (Box 1) and the type of FMG performed can vary between countries and ethnic groups. It can involve the removal of the clitoral glans and/or the labia, or the narrowing of the vaginal orifice with or without the removal of the labia and the clitoral glans.^2^ These practices are prevalent in Eastern and Western Africa, and are becoming increasingly common in high‐income countries owing to migration. More than 200 million girls and women have been subjected to FGM, and an estimated 3 million girls are at risk every year.^3^, ^4^ FGM violates the human rights of women and girls, has no health benefits, and can have significant negative health outcomes.^1^ Evidence‐based guidance to minimize the health consequences of FGM is essential for healthcare providers.^5^

The impact of FGM on obstetric outcomes has been investigated.^2^, ^6^, ^7^, ^8^, ^9^, ^10^ Compared with women without FGM, evidence suggests that women living with FGM have an increased risk of cesarean delivery, postpartum hemorrhage, episiotomy, extended maternal hospital admission, infant resuscitation, and inpatient perinatal death.^8^, ^11^, ^12^, ^13^ The mechanisms of association between FGM and an increased risk for cesarean delivery are unknown, but it has been suggested that this is due to varying amounts of scar tissue.^2^, ^8^ Scar tissue can restrict the vaginal opening but can also cause extensive vaginal and vulvar stenosis, resulting in differing degrees of obstructed labor.^2^, ^6^, ^8^ Scarring can result from FGM itself, or from prior difficult deliveries. FGM has been reported to be associated with difficult deliveries and fetal distress, which can also contribute to increased rates of cesarean delivery.^2^, ^14^

It is also possible that FGM can limit the ability of providers to conduct pelvic exams to assess the safety and feasibility of alternatives to cesarean delivery, such as operative vaginal delivery (forceps or vacuum) or assisted vaginal delivery of a breech fetus. If providers are unable or unwilling to attempt operative vaginal deliveries or breech vaginal deliveries in women with FGM, this could contribute to increased cesarean delivery rates.^2^ It is also possible that providers could have a lower threshold for performing a cesarean delivery in women with FGM owing to concerns about increased risks of complications and lack of evidence to inform clinical decision making. Although several theories exist regarding potential reasons for increased cesarean delivery rates, the indications for cesarean delivery in this population have not been well described or studied previously.

An improved understanding of the reasons for cesarean delivery could help guide obstetric care for women living with FGM, and potentially reduce unnecessary cesarean deliveries. The aim of the present study was to assess whether a clear medical indication was given for cesarean delivery, and to analyze the clinical indications for cesarean delivery based on FGM status. It was hypothesized that any type of FGM would be associated with an increased risk of an unclear medical indication for cesarean delivery.

Box 1 **WHO classification of female genital mutilation.**

**Type I: Partial or total removal of the clitoris^a^ and/or the prepuce (clitoridectomy)**

Type Ia: Removal of the clitoral hood or prepuce only.

Type Ib: Removal of the clitoris^a^ with the prepuce.

**Type II: Partial or total removal of the clitoris^a^ and the labia minora, with or without excision of the labia majora (excision)**

Type IIa: Removal of the labia minora only.

Type IIb: Partial or total removal of the clitoris^a^ and the labia minora.

Type IIc: Partial or total removal of the clitoris^a^, the labia minora, and the labia majora.

**Type III: Narrowing of the vaginal orifice with creation of a covering seal by cutting and apposition the labia minora and/or the labia majora, with or without excision of the clitoris (infibulation)**

Type IIIa: Removal and apposition of the labia minora.

Type IIIb: Removal and apposition of the labia majora.

**Type IV: Unclassified**

All other harmful procedures to the female genitalia for non‐medical purposes, for example, pricking, piercing, incising, scraping, and cauterization.

^a^When total removal of the clitoris is reported, it refers to the total removal of the glans of the clitoris.

## 2 MATERIALS AND METHODS

The present study was a secondary analysis of a WHO multicenter prospective cohort study^8^ examining obstetric outcomes in women in six countries based on different FGM status. Women with singleton pregnancies presenting for delivery at 28 obstetric centers from Burkina Faso, Ghana, Kenya, Nigeria, Senegal, and Sudan between November 1, 2001, and March 31, 2003, were screened for study eligibility. In the parent study, women presenting for an elective cesarean delivery or who were in advanced labor (unable to complete a pelvic exam to determine FGM status prior to delivery) were not eligible. In the present analysis, only patients who had cesarean deliveries were included.

Initial obstetric and medical history data were collected, a physical examination was performed by a trained midwife to determine FGM status and type (Box 1), and patients and neonates were monitored until hospital discharge. Institutional review boards at all participating hospitals and the WHO Secretariat Committee on Research Involving Human Subjects approved the study.

Previous studies^8^, ^15^ have reported different maternal and neonatal obstetric outcomes and estimated healthcare system costs. The focus of the present sub‐analysis was associations between clinical indications for cesarean delivery and FGM status.

Indications for cesarean deliveries were organized into five categories; maternal factors, fetal indications, labor arrest stage 1, labor arrest stage 2, and other. Arrest stage 1 was defined as arrest of cervical dilation prior to achieving full dilation and arrest stage 2 was defined as failure of vaginal delivery after reaching 10‐cm dilation. Indications were then coded by whether the reason given for the cesarean delivery was a commonly report reason for a surgical delivery.^16^ If the reviewing obstetricians (M.I.R. and J.A.) understood the indication given, and the reason was an established indication for operative delivery,^16^, ^17^ it was coded as “a clear medical indication” for cesarean delivery. Otherwise, the cesarean delivery was coded as an “unclear indication.” The classification of indications into the developed categories was reviewed by an independent group of obstetricians not affiliated with the present study.

Descriptive statistics and multinomial logistic regression were used to explore associations between FGM status and indications for cesarean delivery; the data were stratified by whether a clear medical indication for cesarean delivery was provided. Women with any type of FGM were compared with women without FGM using multivariable multinomial logistic regression stratified by whether or not the cesarean delivery was classified as medically necessary. Model covariates included parity, age, urban location, socioeconomic status, and education. Owing to the data being clustered in the six countries, robust standard errors were used to account for clustering.^18^ Statistical analyses were conducted using Stata version 14 (Stata Corp LP, College Station, TX, USA) and variables were compared using the χ^2^ test and the Student *t* test, as appropriate.

## 3 RESULTS

The present study included 1659 women, including 480 who had not undergone FGM, and 1179 who had any type of FGM (Table 1). There were a further three patients living with FGM who underwent cesarean delivery but cesarean‐indication data were not available for these patients. The level of education and the country of residence differed significantly when stratified by FGM status.

**Table 1:** Demographic and pregnancy characteristics (n=1659).^a^

Variable	Patients without FGM (n=480)	Patients with any form of FGM (n=1179)	All patients (n=1659)
Age, y	25.1	26.9	26.4
Country^b^			
Burkina Faso	56 (11.7)	192 (16.3)	248 (14.9)
Ghana	134 (27.9)	94 (8.0)	228 (13.7)
Kenya	24 (5.0)	356 (30.2)	390 (23.5)
Nigeria	164 (34.2)	157 (13.3)	321 (19.3)
Senegal	38 (7.9)	110 (9.3)	148 (8.9)
Sudan	54 (11.3)	270 (22.9)	324 (19.5)
Rural Residence	282 (58.8)	561 (47.6)	843 (50.8)
Education^b^			
No education	159 (33.1)	321 (27.2)	480 (28.9)
Non‐formal	27 (5.6)	126 (10.7)	153 (9.2)
Primary	134 (27.9)	267 (22.6)	401 (24.2)
Secondary	133 (27.7)	329 (27.9)	462 (27.8)
Tertiary	27 (5.6)	136 (11.5)	163 (9.8)
Socioeconomic status			
Low	188 (39.2)	412 (34.9)	600 (36.2)
Medium	278 (57.9)	718 (60.9)	996 (60.0)
High	14 (2.9)	49 (4.2)	63 (3.8)
Multiparous	250 (52.1)	669 (56.7)	919 (55.4)
Clear medical indication for cesarean delivery^c^	382 (79.6)	912 (77.4)	1294 (78.0)
Categorization of cesarean delivery indication			
Maternal factors	65 (13.5)	222 (18.8)	287 (17.3)
Fetal factors	158 (32.9)	344 (29.2)	502 (30.3)
Labor arrest stage 1	51 (10.6)	391 (33.2)	151 (9.1)
Labor arrest stage 2	159 (33.1)	391 (33.2)	550 (33.2)
Other	47 (9.8)	122 (10.3)	169 (10.2)

Abbreviation: FGM, female genital mutilation.

a Values are given as mean or number (percentage).

b *P*<0.001.

c *P*=0.320.

The frequency of verbatim indications given by healthcare providers for cesarean deliveries, how these indications were classified, and whether these were classified as clear medical indications were collated (Table 2). Indication data were missing for 3 (0.2%) patients. There were 66 different primary indications for cesarean delivery recorded; the most commonly recorded factors were any type of labor arrest and fetal factors.

**Table 2:** Indications for cesarean delivery

Category of indication and specific indication for cesarean delivery	Clear medical indication for cesarean delivery provided?	Value^a^
Maternal factors		287 (17.3)
Uterine prolapse	No	133
Ruptured uterus	Yes	29
Pregnancy‐induced hypertension	No	24
Pre‐eclampsia/toxemia	No	21
Hypertension	No	19
Imminent eclampsia	Yes	17
Intrapartum hemorrhage	Yes	15
Intrapartum eclampsia	Yes	11
To save mother's life	Yes	10
Failure to progress/Maternal distress	Yes	4
Amniotic infection	No	3
Toxemia	No	1
Fetal factors		502 (30.5)
Fetal distress	Yes	303
Breech presentation	Yes	56
Malpresentation	Yes	33
Cord prolapse	Yes	29
Transverse lie	Yes	25
Big baby (large baby)	Yes	13
Face presentation	No	13
Fetal macrosomia	Yes	9
Arm prolapse	No	8
Brow presentation	Yes	5
Hydrocephalus	Yes	3
Uterine hypercontractility	Yes	2
Retained second twin	Yes	2
Sizeable fetus	No	1
Labor arrest Stage 1		103 (6.2)
Failed induction	Yes	57
Cervical dystocia	Yes	29
Failure of dilation	Yes	8
Cervical diaphragm	No	6
Delayed first stage	Yes	1
Pinhole cervix	No	1
Dilation	No	1
Labor arrest Stage 2		550 (33.1)
Positional CPD	Yes	203
Obstructed labor	Yes	99
Contracted pelvis	Yes	69
Prolonged labor	Yes	55
Fetopelvic disproportion	Yes	48
Small pelvis	No	39
Obstructed prolonged parturition	Yes	9
Delayed second stage	Yes	8
Vaginal stenosis	Yes	8
Atonic uterus	Yes	8
Failure to progress	Yes	3
Prolonged 2nd stage	Yes	1
Other		215 (12.9)
Failure to progress in trial of scar	Yes	45
Abruptio placenta	Yes	36
Placenta previa	Yes	28
Previous cesarean delivery	Yes	19
Premature rupture of membranes	No	18
High head	No	18
Bad obstetrics history	No	17
Vasa previa	Yes	11
Couppres^b^	No	4
Hematoma placental	No	3
Post date	No	3
Bicornuate	No	3
Multiple vaginal warts	No	1
Intrauterine growth restriction	No	2
Grand multiple	No	1
Persistent occipito posterior position	No	2
Fibroid in pregnancy	No	1
Fetal prematurity	No	1
Meconium stained liquor in previous	No	1
Cervical cyst	No	1
Missing data		3 (0.2)
Total		1662

a Values are given as number (percentage) or number.

b “Couppres” was the indication provided in the original hostpial data; no definition was available.

Among patients with no type of FGM, 382 (79.6%) cesarean deliveries had a clear medical indication recorded; this did not differ significantly from the 912 (77.4%) patients with any type of FGM (*P*=0.320).

The results of the bivariate and multivariable models were similar so only the multivariable model was included in the present manuscript. Among women with unclear indications for cesarean delivery, women living with FGM had an increased risk of undergoing cesarean delivery for maternal factors (adjusted relative risk ratio [aRRR] 3.92, 95% confidence interval [CI] 1.30–11.71), stage 1 arrest (aRRR 7.74, 95% CI 1.33–45.07), stage 2 arrest (aRRR 6.63, 95% CI 3.74–11.73), and other (aRRR 2.41, 95% CI 1.04–5.60) factors rather than fetal factors compared with patients without FGM (Table 3). By contrast, among women who had clear medical indications for cesarean delivery, no associations were observed between indication category and FGM status. Among women living with type 3 FGM, a trend towards increased risk on non‐fetal indication categories was observed but, owing to the limited sample size, all FGM types were combined (data not shown).

**Table 3:** Multinomial logistic regression of relative risk ratios of cesarean delivery indication categories (n=1659).^a^

Variable	Maternal versus fetal factors (ref)	Stage 1 arrest versus fetal factors (ref)	Stage 2 arrest versus fetal factors (ref)	Other versus fetal factors (ref)
Unclear medical indication for cesarean delivery				
FGM status				
Patients without FGM	1.00	1.00	1.00	1.00
Patients with any type of FGM	3.92 (1.31–11.71)	7.74 (1.33–45.07)	6.63 (3.74–11.73)	2.41 (1.04–5.60)
Obstetric characteristics				
Nulliparous	1.00	1.00	1.00	1.00
Multiparous	1.27 (0.73–2.20)	1.27 (0.73–2.20)	1.14 (0.41–3.15)	1.30 (0.68–2.47)
Demographic characteristics				
Age, y	0.98 (0.93–1.02)	0.93 (0.85–1.03)	0.85 (0.80–0.90)	1.01 (0.92–1.11)
Education (vs none)	2.30 (1.48–3.57)	2.29 (1.48–3.56)	1.06 (0.67–1.66)	1.43 (0.95–2.15)
Socioeconomic status (vs low)	0.42 (0.09–1.77)	0.41 (0.01–1.77)	0.40 (0.67–1.66)	0.33 (0.07–1.56)
Rural residence (vs urban)	1.37 (0.33–5.77)	0.73 (0.15–3.67)	1.30 (0.36–4.70)	0.81 (0.29–2.26)
Clear medical indication for cesarean delivery				
FGM status				
Patients without FGM	1.00	1.00	1.00	1.00
Patients with any type of FGM	1.46 (0.75–2.81)	0.71 (0.39–1.32)	1.00 (0.59–1.72)	1.15 (0.70–1.89)
Obstetric characteristics				
Nulliparous	1.00	1.00	1.00	1.00
Multiparous	1.60 (0.48–5.27)	1.32 (0.71–2.43)	0.92 (0.61–1.40)	4.95 (2.85–8.59)
Demographic characteristics				
Age, y	1.01 (0.95–1.06)	1.00 (0.95–1.06)	0.98 (0.94–1.02)	1.00 (0.96–1.05)
Education (vs none)	0.93 (0.77–1.13)	0.93 (0.77–1.13)	0.72 (0.56–1.07)	0.85 (0.63–1.41)
Socioeconomic status (vs low)	1.30 (0.74–2.27)	1.30 (0.74–2.27)	1.15 (0.83–1.60)	0.82 (0.46–1.45)
Rural residence (vs urban)	0.62 (0.43–0.88)	0.62 (0.43–0.88)	0.78 (0.56–1.07)	0.74 (0.41–1.31)

Abbreviation: FGM, female genital mutilation.

a Values are given as adjusted relative risk ratios (95% confidence intervals).

## 4 DISCUSSION

Many studies have described increased cesarean delivery rates among women living with FGM; however, little evidence exists to explain the mechanisms that contribute to these increases. The findings of the present study suggest that among women with an unclear medical indication for cesarean delivery, women living with FGM are significantly more likely to have a cesarean delivery owing to a maternal factor or arrest disorder.

There were limitations to the present study that should be considered when interpreting the findings. The study did not include women who had scheduled cesarean deliveries, only those who had a trial of labor. It is possible that, at some sites, women with the most extensive FGM are advised to have a scheduled cesarean; this would bias the results of the present study toward the null hypothesis. Further, the distinction between clear and unclear medical indications was not based on a validated measure, but expert opinion. To address this limitation, a sensitivity analysis for the most common indication that was unclear was conducted, and the magnitude and significance of the findings persisted (data not shown). Additionally, the indication categories for cesarean deliveries were not based on a validated measure but instead were based on the primary reason recorded by the surgeon for the cesarean delivery only. A conservative estimate was included of categories pertaining to obstructed labor, by considering fetal malpresentation and macrosomia separately. These factors could also have potentially contributed to stage 1 or stage 2 labor arrests. Multiple factors can contribute to the decision to perform a cesarean delivery and the present study only examined the primary reason given. Despite these limitations, the study had several important strengths. The data used were collected from 28 centers across six African countries and the sample size was, to the best of our knowledge, the largest used to provide information on why women with FGM have cesarean deliveries and important insights into the care of women living with FGM.

Provider practices could explain the results obtained. It is possible that providers have a lower threshold for performing cesarean deliveries in women living with FGM, in particular type 3, out of concern for maternal well‐being, a lack of training in how to perform defibulation, and minimal evidence to help guide clinical decision making. An inability to perform a pelvic examination adequately would impact on labor assessment, and this could also influence the threshold for deciding to perform a cesarean delivery for women with FGM. The obstetric care of women living with FGM requires specialized knowledge and skills to minimize morbidity and maximize health outcomes for patients and their children.^5^ Currently, FGM is not included in the curriculum of most medical, nursing, midwifery, and public health training programs and recommendations about clinical management, defibulation, and legislation surrounding FGM are not well known. The recently published WHO evidence based guidelines^19^ for healthcare providers caring for women living with FGM are expected to support efforts to enhance the training of healthcare providers in these aspects.

Multiple factors beyond the anatomic changes associated with FGM could likely contribute to frequent cesarean deliveries being performed more frequently with reduced indications. The present study included adjustments for socioeconomic status, education, and rural residence, demographic factors that appeared likely to influence the results, and the observed associations persisted. The present study also explored the categorization of unclear and clear medical indications further; this included conducting a sensitivity analysis to determine if prolapse (the most commonly recorded category considered an unclear indication) was driving the observed associations; the findings remained the same after omitting patients with uterine prolapse (data not shown).

Other unobserved confounders could partially explain the associations observed. Women living with FGM could present late for delivery care. A mixed‐methods study of Somali immigrants in the USA^20^ demonstrated that some women with FGM delay seeking medical attention until labor is well advanced out of a concern they will be forced to have a cesarean delivery. The present study did not control for the duration of pregnancy among patients who presented for prenatal care or the timing of defibulation, which could also impact the cesarean delivery rate.

Cesarean delivery is a life‐saving intervention for women and neonates when performed for medically indicated reasons.^21^ However, it is a major surgery that can be associated with significant morbidity and even mortality.^22^ The overall rate of cesarean delivery in the parent study population was low, 6%,^8^ and was below the rate at which decreases in maternal and neonatal mortality rates are observed at the population level.^21^, ^23^, ^24^ It is possible that even a low rate of elective or scheduled cesarean deliveries could explain these findings; this was a limitation. When the primary indications within the maternal indications category were explored in more detail, approximately one‐third of indications were for hypertensive disorders and nearly half were for uterine prolapse, with a smaller proportion of cesarean deliveries indicated for hemorrhage‐related emergencies. Hypertensive disorders, individually, are not solely a reason for cesarean delivery and it is likely that secondary factors contributed to cesarean delivery decisions for these patients. An improved understanding of provider decision making regarding delivery could help to reduce unnecessary cesarean deliveries among women living with FGM.

The elimination of FGM is a key focus of the sustainable development goal to achieve gender equality and empower for all women and girls.^25^ Ending FGM will take a systematic and intense effort at multiple levels and it is essential that effective policies and interventions to eliminate FGM are identified and implemented. Equally important is the need for health evidence and training to minimize the negative health outcomes for girls and women living with FGM.^5^ WHO is working to address this gap in the evidence through new research and guidelines for healthcare providers. Full implementation of evidence‐based care and practice guidelines is critical to improving the health of girls and women living with FGM.

The present study provided important information on the reasons patients with FGM have cesarean deliveries, highlighting the need for improved provider training and research to guide the healthcare of women living with FGM. These findings have significant policy and practice implications. Multiple factors at the provider, patient, and system level likely influence the increased risk of potentially unnecessary cesarean deliveries. Further work is needed to understand how women living with FGM are treated during pregnancy and delivery. Scant data exist to guide the medical care of these patients.^5^ Evidence is needed to guide the medical care of women living with FGM, in particular training for healthcare providers in the specific healthcare needs of this population. Data to help guide patients and healthcare providers in both accepting and offering defibulation could help mitigate the obstetric consequences of FGM.

## AUTHOR CONTRIBUTIONS

MIR contributed to the conception of the study and the categorization schema, performed data cleaning, and contributed to writing the manuscript. LS contributed to the conception of the study and writing the manuscript. JA contributed to the development of the categorization schema and writing the manuscript. MJH performed data cleaning, designed and performed the analyses, and contributed to writing the manuscript.

## CONFLICTS OF INTEREST

The authors have no conflicts of interest.
